# Psychometric properties and measurement invariance of the Rigid and Flexible Persistence Scale in a Brazilian professional sample

**DOI:** 10.1186/s41155-022-00240-0

**Published:** 2023-01-03

**Authors:** Evandro Morais Peixoto, Marcus Vinicius Silva, Ana Paula Porto Noronha, Tanya Chichekian, Robert J. Vallerand

**Affiliations:** 1 University of São Francisco, USF-105 Waldemar César da Silveira St, Jardim Cura D’ars, Campinas-SP, 13045-510 Brazil; 2 Université de Sherbrooke - Campus de Longueuil, 150, Place Charles-Le Moyne, Longueuil, Québec J4K 0A8 Canada; 3 Université du Québec À Montréal Station Centre-Ville, Montreal, QC H3C 3P8 Canada

**Keywords:** Positive psychology, Organizational psychology, Passion, Persistence, Occupational self-efficacy

## Abstract

Persistence involves the intention to maintain efforts when faced with obstacles and challenges, in order to achieve a specific goal. The Rigid and Flexible Persistence Scale (RFPS) is a self-report measure that assesses persistence that is theoretically derived from the premises of the Dualistic Model of Passion. The aim of the present research was to adapt the RFPS to Brazilian Portuguese and to estimate its evidence of validity and reliability in the context of work. Participants were 400 professionals, 55.8% women, aged between 18 and 68 years. The results indicate that the RFPS presented validity evidence based on the content. Corresponding to the theoretical expectations, exploratory and confirmatory factor analyses demonstrated adequacy of the structure composed of two factors, explaining 75% of the data variance, with good levels of reliability. The RFPS also revealed strong invariance across gender and work conditions (in-person vs remote). Flexible persistence showed positive association with harmonious passion and occupational self-efficacy. Conversely, rigid persistence showed positive association with obsessive passion. It was observed a mediational role of occupational self-efficacy in the relationship between harmonious passion and flexible persistence. Overall, the findings suggest that the RFPS is an adequate measure of persistence in a Brazilian occupational sample.

## Introduction

Aiming to consider healthy and potential issues of individuals, Positive Psychology has focused on topics such as optimism, well-being and character strengths, demonstrating the importance of acting in both the promotion of mental health and the prevention of risks (Seligman, 2019a). In this perspective, researchers have been investigating the way people related to work through variables such as persistence (Vallerand et al., 2022). However, in the Brazilian context, there is still a scarcity of studies aimed at understanding the effects of persistence in the work environment. One of the aspects that contribute to this gap in the area is the lack of instruments with psychometric properties that ensure the operationalization of this construct.

Historically, the level of continuous engagement to achieve a goal has been considered the main characteristic of persistence (Gimeno et al., 1997). However, qualitative differences can affect the way in which the persistence is manifested. This could take a dualistic form such that an individual would persist in either an adaptive fashion (flexible persistence) or a less adaptive way (Rigid Persistence) toward the pursuit of an objective (Chichekian & Vallerand, 2022; Vallerand et al., 2022). Considering that this research has been conducted in North America, a culture where persistence has great appeal, further study is needed to access whether it can be applied to other cultures as well (Vallerand et al., 2022).

## Persistence

Persistence is typically defined as a characteristic of intentional behavior in which persisting is synonymous with effort directed towards achievement, despite fatigue (McDougall, 2013). This construct is also considered a continuous, voluntary and goal-directed force, despite discouragement and the presence of obstacles (Peterson & Seligman, 2004). Persistence has also been included as part of self-control, as it refers to non-cognitive factors such as tenacity and perseverance. Therefore, persisting implies maintaining efforts towards a specific goal and prioritizing activities related to these goals, to the detriment of probable distractions and other alternatives (Farrington et al., 2012).

From this perspective, it can be stated that high levels of persistence are considered more desirable, as they allow for higher levels of optimism (Duckworth et al., 2007), motivation (Atkinson & Cartwright, 1964; Peterson, 2000) and satisfaction with the activity (Sheldon and Houser-Marko, 2001; Seligman, 2019b). However, qualitative differences can impact the way people persist toward a goal that go beyond persisting versus letting go of a goal (Wrosch et al., 2003). Accordingly, Vallerand et al. (2022) propose the existence of two types of persistence: Rigid Persistence (RP), which is characterized by continuously seeking the achievement of a goal irrespective of conditions, and flexible persistence (FP), by which the individual maintains goal seeking while keeping harmony with other life goals, making it possible to achieving both activity and other life goals.

In order to improve the comprehension of the qualities of persistence, the Rigid and Flexible Persistence Scale (RFPS) was developed by Vallerand et al. (2022), in the Canadian context, in order to operationalize the two-dimensional structure and the association of expressions of this construct with the types of passion, and to differentiate persistence from passion. In the first study on the RFPS, the instrument was used with a sample of 512 workers from various occupations. Results provided support for the dual structure of the scale through exploratory and confirmatory factor analyses, as well as good reliability level.

In addition, other results (study 1) provided support on the role of passion as a determinant of the two types of persistence. Specifically, the results from structural equation modeling suggest that obsessive passion positively associated rigid persistence (β = 0.54, *p* < 0.001) and negatively associated flexible persistence (β =  − 0.30, *p* < 0.001), whereas harmonious passion was related with flexible persistence (β = 0.51, *p* < 0.001) and, to a lesser extent, rigid persistence (β = 0.13, *p* = 0.005). Of interest, the two types of persistence were found to predict different consequences. Specifically, both RP and FP were found to predict adaptive outcomes within the confines of the activity at hand (e.g., work), whereas only FP was found to predict adaptive life outcomes (e.g., wellbeing) outside of the activity (Vallerand et al., 2022).

The above dual definition of persistence introduces new ideas about the nature, determinants and consequences of persistence and assumes that passion for an activity can contribute to engagement and persistence in that activity (Chichekian & Vallerand, 2022, Vallerand, 2015; Vallerand et al., 2003). As proposed by Vallerand et al. (2003), passion can be defined as a strong inclination towards an activity of interest to the person, in which they invest a significant amount of time and energy for its accomplishment. This relationship with the activity can be expressed in two ways, harmonious passion (HP) and obsessive passion (OP), which generate, each in their own way, a strong commitment to the activity. Thus, HP results in a type of persistence that is richer and more full, therefore more flexible, than OP, which leads to a more rigid persistence (Vallerand et al., 2019; Vallerand et al., 2022).

The above underscores the importance of measuring the two different types of persistence, since each dimension leads to different psychological consequences. Even though both persistence types can contribute to engagement and performance at work, rigid persistence may lead to higher levels of burnout, stress, and anxiety, while flexible persistence may lead to higher levels of job satisfaction and well-being. Assessing these characteristics would help in the development of organizational strategies to promote the health of employees, as well as prevent risks.

Concerning persistence determinants, Bandura (1995) emphasized the relevance of self-efficacy, since the belief that a subject would have regarding their ability to perform and manage the actions necessary to complete certain tasks (such as work) would influence the degree to which they would or would not persist in carrying out this activity. The study by Feng and Chen (2020) observed the mediating effect of entrepreneurial self-efficacy on the relationship between passion and persistence to undertake and perform in business. However, it is worth mentioning that this study simply used persistence as a unidimensional variable.

Considering that in Brazilian context tools accessing both forms of persistence do not exist. The need for the adaptation of an instrument capable of capturing the two dimensions of persistence is important. This adaptation could lead to the conduct of new studies to comprehend the potential of this model for adaptive or maladaptive involvement in the achievement of objectives in the context of work. In order to contribute to filling this gap, the aims of the present study were to adapt the Rigid and Flexible Persistence Scale (RFPS) in the Brazilian Portuguese language and estimate its validity evidence based on content, internal structure, and on relationship with external variables, namely, passion for work and occupational self-efficacy.

The present study was based on the hypothesis that the content of the Brazilian Portuguese RFPS would yield psychometric properties equivalent to the original scale (H1) and that a structure composed of two correlated factors would be found in Brazilian samples (H2). Flexible persistence was also expected to present a moderate positive correlation with harmonious passion and a moderate negative correlation with obsessive passion (H3). Furthermore, rigid persistence was expected to be positively correlated with obsessive passion and, to a lesser extent, with harmonious passion (H4). Hypothesis 5 was that there would be a positive and moderate relationship between occupational self-efficacy and flexible persistence. However, there would be a null relationship between rigid persistence and occupational self-efficacy. Finally, in line with Feng and Chen (2020), the conceptual model was tested in which occupational self-efficacy was positioned as a mediating variable in the relationship between passion and persistence (H6).

## Method

### Participants

A non-probabilistic sample was composed of 400 Brazilian workers (55.8% female), 18 to 68 years of age (*M* = 35.6; SD = 10.5). Individuals were from the Southeast (68.5%), South (19.5%), Northeast (4.8%), Central-west (5.5%), and North (1.7%) of the country. In addition, they were attending or had completed postgraduate studies (54.0%), undergraduate (37.8%), high school (7.5%), or elementary education (0.7%). The professionals held different positions in which they had been for a mean of 64.8 months (SD = 82.9; approximately 5.4 years) and worked a mean of 33.1 h (SD = 17.3) per week in private (62%) or public (32.5%) institutions or autonomously (5.5%), in person (59.7%) or remotely (40.3%).

### Instruments

#### *The Rigid and Flexible Persistence Scale (*Vallerand et al., 2022*)*

Initially constructed in English, eight items constitute the scale that assesses persistence in two dimensions specific to the activity, in this case work: rigid persistence (e.g., I am willing to do anything to reach the top at work/*Estou disposto a fazer qualquer coisa para chegar ao topo no trabalho*.) and flexible persistence (e.g., I work hard at my work goals, but other things matter as well/*Eu trabalho duro nos objetivos dos meu trabalho, mas outras coisas também são importantes*.). The instrument is of the self-report type, in which participants must indicate how much they agree with each statement using a seven-point Likert-type scale, in which 1 corresponds to “do not agree at all” and 7 to “very strongly agree”. Results of both exploratory and confirmatory factor analyses supported the dual structure of the scale reflecting rigid and flexible persistence. The internal consistencies of the dimensions of the original scale (Vallerand et al., 2022, study 1) showed adequate reliability, indicated by Cronbach’s alpha coefficient, 0.85 for rigid persistence and 0.76 for flexible persistence.

#### *The Passion Scale (*Vallerand et al., 2003*)*

The Passion Scale has been fully validated and shown to possess high levels of validity and reliability (see Marsh et al. 2013; Vallerand and Rahimi, in press; Vallerand et al., 2003). Adapted to the Brazilian context by Peixoto et al. (2019), the scale was used to measure passion for work. It consists of 12 items, six for harmonious passion (*e.g.* My work is in harmony with other activities in my life/*Meus trabalho está em harmonia com as outras atividades em minha vida.*) and six for obsessive passion (e.g. I have difficulties controlling my urge to work/*Tenho dificuldade em controlar minha necessidade em desempenhar o meu trabalho*.) answered on a Likert-type scale ranging from 1 (strongly disagree) to 7 (strongly agree). Internal consistency showed adequate reliability both dimensions, harmonious passion (α = 0.90) and obsessive passion (α = 0.86). The two-factor model showed adequate fit for instrument’s internal structure (χ^2^/df) = 3.96, TLI = 0.94, CFI = 0.96, RMSEA = 0.08, AIC = 23,992.86, BIC = 24,225.10).

#### Occupational Self-efficacy Scale–Short form (OSS-SF; Rigotti et al. 2008)

The OSS-SF refers to the reduced form of the OSS (Schyns and von Collani, 2002). Translated and adapted to the Brazilian context by Damásio et al. (2014), it was named *Escala de Autoeficácia Ocupacional – Versão Reduzida*. The instrument consists of six items (e.g. I meet the goals that I set for myself in my job/*Eu alcanço as metas que eu estabeleço para mim mesmo em meu trabalho*.), which are answered on a Likert-type scale, ranging from 1 (strongly disagree) to 6 (strongly agree). The single-factor structure was demonstrated by means of confirmatory factor analysis (*χ*^2^/df = 3.45, SRMR = 0.05, RMSEA = 0.09, CFI = 0.98, TLI = 0.95, CAIC = 461.669), and internal consistency showed adequate reliability (α = 0.78).

### Procedures

#### Cross-cultural adaptation

With the authorization of the author of the original version, Robert J. Vallerand, the RFPS was independently translated from English into Portuguese by four bilingual professionals (namely, two psychologists, a professor and an English teacher). Subsequently, a synthesis version was obtained by an expert committee composed of four researchers specializing in psychological assessment and psychometrics. In a subsequent step the instrument was analyzed based on four criteria: clarity of language, practical relevance, theoretical relevance, and theoretical dimensions by three judges that were experts in Organizational and Work Psychology. A group composed of five professionals, two doctors, a salesperson, a seamstress and a process engineer (60% male), aged between 25 and 37 years (*M* = 30; SD = 4.58) was accessed to evaluate the scale content intelligibility. None of the participants had difficulty interpreting the instructions or items of the instrument since the RFPS had clearly and objectively drafted items. Finally, the Brazilian version of the scale was back-translated into English by a native translator, which made it possible to verify the equivalence between the content of the original and the Brazilian version.

#### Ethical aspects

The project was submitted to the Human Research Ethics Committee, and after approval the instruments were compiled on the Google Forms platform. Due to the COVID-19 pandemic data was collected online. The link to answer the online instruments was sent to participants from social networks. The participants who agreed to participate initially completed the consent form. Subsequently, the participants responded to the sociodemographic questionnaire, the Persistence Scale, the Passion Scale, and the Occupational Self-efficacy Scale–Short form taking approximately 15 min.

#### Data analysis

The content validity coefficient (CVC) was used to verify evidence of validity based on the content of the instrument, considering values above 0.70 (Hernandez-Nieto, 2002) regarding language clarity, practical relevance, and theoretical pertinence. In relation to the theoretical dimensions, the Kappa coefficient, considering values above 0.60, was used to evaluate the level of agreement among the experts (Fleiss, 1981).

The analyses directed toward evidence of validity based on internal structure, reliability and the relationship with an external variable were performed using the statistical software Factor (Lorenzo-Seva & Ferrando, 2006), Mplus 7.3 (Muthén & Muthén, 2017), and Jamovi (2020). It should be noted that the participants were randomly divided into two groups. Sample 1 (*n* = 150) was used to verify the factor structure through EFA, while sample 2 (*n* = 250) was selected for the CFA. In spite of the extensive and inconclusive debate over sample size in factor analysis, both samples could be considered adequate as they fit most of the classical rules-of-thumb suggested in the literature, as subject to item ratios of 10:1 and 10–20:1, or number of observation greater than 100 (Hair et al., 2010). However, it was decided to have a greater number of observations to carry out the CFA, which, as it is a restrictive model, allows the estimation of the loads of the items that represent the factor, forcing the factor loads of the items that were not designed to represent them to 0, which can make the model too restrictive to the point of harming the fit indicators (Marsh et al., 2020; Morin et al., 2020).

Exploratory factor analysis (EFA) was performed using the statistical software Factor, with a parallel analysis method, as an indicator for the retention of the factors, polychoric correlation, diagonally weighted least squares (DWLS) estimation, and Promin oblique rotation (Hongyu, 2018; Lim & Jahng, 2019). To verify the reliability level of the internal consistency, Cronbach’s alpha and McDonald’s omega were used (George & Mallery, 2002). According to the literature, indices greater than 0.70 are considered good indicators of reliability (Tabachnick & Fidell, 2019).

The CFA was performed using the Mplus software with the weighted least squares mean and variances adjusted (WLSMV) estimation method. The fit of the measurement model to the data was evaluated using the indices recommended by Muthén and Muthén (2017), namely, χ^2^/df, RMSEA, CFI, and TLI. The reference values commonly used in the specialized literature were used as adequacy parameters: χ^2^/df < 5, RMSEA < 0.08, CFI and TLI > 0.90 (Tabachnick & Fidell, 2019). Finally, the RFPS scale’s generalizability was assessed with participants from both samples. More precisely, using invariance testing we assessed whether the RFPS was free of measurement bias across gender and work conditions (i.e., presential and remote). Thus, fit indices are estimated for the different invariance models (configural, metric, and scalar). The configural model is evaluated with the same parameters as the AFC, the adequacy of these indices indicates the equivalence of the factor structure across groups. In order to evaluate the subsequent models, it is necessary to examine the extent to which the invariance constraint between the groups contributes to variability in the fit indices, being considered indicators of non-invariance ΔCFI ≥  − 0.01, ΔRMSEA ≥ 0.015, ΔSRMR ≥ 0.01 (or 0.03 in metric invariance) (Chen, 2007; Cheung and Rensvold, 2002).

To verify the validity based on the relationship with other variables, Pearson’s coefficient (*r*) was calculated, using the Jamovi software, to assess the degree of correlation between the constructs and their directions (whether positive or negative). Significance levels of *p* < 0.05 were considered. The magnitudes of the correlations were interpreted according to Cohen’s classification (1988): from − 0.09 to 0.09 null; from 0.10 to 0.29 small; from 0.30 to 0.49 medium and from 0.50 to 1.0 large. Finally, to evaluate the mediation model, structural equation modeling was carried out, using multiple regressions between the latent variables estimated from the items of the respective instruments. These analyses were also carried out using the Mplus 7.3 software and the goodness of fit of the data to the theoretical model was verified through the fit indices described in the CFA. To assess the significance of the total, direct, and indirect effects, a significance level of *p* < 0.05 was adopted, with the 95% confidence interval (CI) estimated through a bootstrap procedure, a method that allows for more robust estimates for the confidence limits of these effects, as well as for estimates of the standard errors (SE) associated with these statistics. Using the a priori sample size calculator for Structural Equation Models (Soper, 2022), that considering the number of latent (5) and observed variables included in the mediational model presented in this study (26), a power analysis of 80%, probability level of 0.05, and the effect size of 0.20 recommended a minimum sample of 376 participants (Westland, 2010), suggesting the adequacy of the available sample.

## Results

### Content-based evidence of validity

The RFPS was analyzed based on four criteria: language clarity (LC), practical relevance (PR), theoretical relevance (TR) and theoretical dimension (TD) through the content validity coefficient (CVC) procedure (Hernandez-Nieto, 2002). Regarding the LC, 75% of the items obtained CVCc equal to 0.90. Just two items (“I work hard at my work goals, but other things matter as well.” and “I work hard to achieve a work goal, but can stop if necessary.”) had scores lower than 0.70 (CVCc = 0.63). This fact can be justified by the way both items are written, presenting two ideas. However, in theory it is necessary for the writing to provide information that reflects persistence (Vallerand et al., 2022). Therefore, the total CVC of this criterion was 0.83, indicating that the cutoff point suggested by the literature was exceeded (Hernandez-Nieto, 2002). Regarding the PR aspect, 100% of the items scored above 0.70 (CVCc = 0.83 to 0.90). The total CVC for this item was 0.86. Regarding TR, 100% of the items presented CVCc values between 0.76 and 0.90. In general, the theoretical relevance of the items showed evidence of content validity, since the total CVC of this aspect was 0.85. Finally, all items obtained 100% agreement regarding the flexible or rigid persistence theoretical dimensions.

#### Factorial validity: internal structure and reliability

At first, using the Factor software, the EFA was performed with sample 1. In a preliminary analysis it was observed that there were no missing values on the database, the factorability of the data matrix was verified using the Kaiser–Meyer–Olkin criteria (KMO = 0.67) and Bartlett’s test of sphericity (*χ*^2^ (28) = 554.5; *p* < 0.0001), indicating the possibility of performing EFA. Accordingly, the parallel analysis suggested the retention of two factors, since both presented values higher than those observed in the factors allocated in the 95% percentile of the randomly estimated data (item 1 = 30.06%; item 2 = 24.33%). Table 1 presents the model estimated through EFA with the factor loadings and the commonality indices of the items, as well as the percentage of explained variance and reliability indicators.

**Table 1:** Exploratory factor analysis for the items of the Rigid and Flexible Persistence Scale (*n* = 150)

Items	FP	RP	*h*^2^
1. I work hard at my work goals, but other things matter as well	**.975**	− .188	.931
2. I work hard to achieve a work goal, but can stop if necessary	**.544**	− .001	.295
3. I really focus on my work when it's time to do it	**.585**	.089	.359
4. I try to reach my work goals but not at the expense of other life goals	**.836**	− .064	.689
5. I am willing to do anything to reach the top at work	.135	**.741**	.617
6. When it comes to reaching my goals at work, nothing else matters	-.058	**.913**	.823
7. It is OK for me to focus only on work goals in order to succeed	.066	**.816**	.653
8. I am willing to let go of some things in life in order to excel at work	.114	**.492**	.325
% Explained variance	41.65	33.35	
Total % explained variance	75.00		
Correlation between factors	.148		
Cronbach’s alpha	.81	.82	
McDonald’s omega	.82	.83	

*FP* Flexible persistence, *RP* Rigid persistence, *h*^2^ Communalities

The correlation between the two factors was positive, significant, but low (*r* = 0.148, *p* < 0.05). The factor loadings of the items varied between 0.492 (item 8) and 0.975 (item 1), and the model was able to explain 75% of the total variance of the data. Regarding the reliability of the RFPS indicators, both the FP factor (ω = 0.82; α = 0.81) and the RP factor (ω = 0.83; α = 0.82) presented adequate levels of internal consistency. Additionally, good fit indices were observed for the saturated model (*χ*^2^ (df) = 23.039 (13); NNFI = 0.96; CFI = 0.98; RMSEA = 0.07, 95% CI [0.000–0.0972]).

**Fig. 1:**
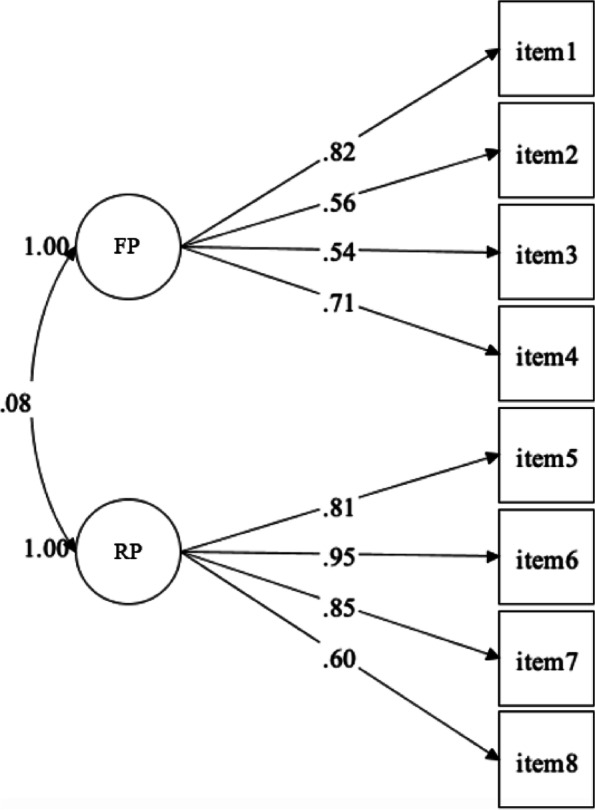
Confirmatory factor analysis for the items of the Rigid and Flexible Persistence Scale (*n* = 250). Note. All factor loadings were statistically significant (*p* < .001)

Confirmatory factor analysis (CFA) was performed using the statistical software Mplus, with WLSMV estimation, with the data from 250 participants (sample 2), also it was observed that there were no missing values on the database. Satisfactory fit indices were found for the model (*χ*^2^ (df) = 27.824 (19); CFI = 0.97; TLI = 0.96; RMSEA = 0.04; 90% CI [0.000–0.075]), as well as adequate levels of reliability for the factors (FP, ω = 0.73; α = 0.71; RP, ω = 0.86; α = 0.85). The factor loadings of items 1, 2, 3, and 4 corresponding to FP ranged from 0.54 to 0.82. Items 5, 6, 7, and 8 related to RP had factor loadings between 0.60 and 0.95. Additionally, there was a correlation of low magnitude between the factors (*r* = 0.08; *p* = 0.222), and Fig. 1 illustrates these results.

These findings provide the first evidence of validity based on the internal structure and reliability of the Brazilian version of the RFPS in the context work and organizational psychology. Finally, the psychometric model invariance across gender and work model (presential and remote) was verified with both samples. The results are presented in Table 2.

**Table 2:** Invariance models

**Models**	*χ*^**2**^** (df)**	**CFI**	**TLI**	**RMSEA**	**90% CI**	**ΔCFI**	**ΔTLI**	**ΔRMSEA**
Invariance across genders								
Configural invariance	84.526 (38)	.985	.979	.078	.056–.100			
Metric invariance	95.169 (44)	.984	.980	.076	.055–.097	− .001	.001	− .002
Scalar invariance	144.936 (82)	.980	.987	.062	.045–.078	− .004	.007	− .014
Invariance across work conditions (in person and remote)								
Configural invariance	98.686 (38)	.982	.974	.090	.068–.112			
Metric invariance	107.790 (44)	.981	.976	.085	.065–.106	− .001	.002	− .005
Scalar invariance	142.863 (82)	.982	.982	.071	.044–.078	.001	.006	− .014

*χ*^**2**^ Chi-squared, *df* Degrees of freedom, *CFI* Comparative fit index, *TLI* Tucker–Lewis index, *RMSEA* Root mean square error of approximation, *CI* Confidence interval, Δ The difference between more restrict and reference model

The results suggest strong equivalence of the measurement model across groups since there was no decrease in the fit indices exceeding the literature recommended cut-offs, as the model restriction increased. Once this evidence was estimated, the validity evidence based on the relationship with an external variable was evaluated considering the relationship between persistence, passion for work, and occupational self-efficacy. The results are shown below.

#### Construct validity based on relationships with external variables

Using the Jamovi software, correlation analyses and structural equation modeling were performed with the entire sample of 400 participants. According to Table 3. Occupational Self-Efficacy presented moderate positive correlation with FP and weak positive correlation with RP. FP exhibited a moderate magnitude positive correlation with HP. RP was shown to be positively correlated, with a moderate magnitude, with HP and to a greater extent with OP. Finally, the correlation between the persistence factors was positive and weak.

**Table 3:** Correlations among the dimensions of passion, occupational self-efficacy, and persistence

	FP	RP	HP	OP	OS
FP	—	0.088	0.542***	-0.061	0.509***
RP	.129**	—	0.276***	0.627***	0.122*
HP	.420***	.341***	—	0.353***	0.545***
OP	− .042	.516***	.401***	—	0.106*
OS	.399***	.132***	.506***	.109*	—

*FP* Flexible persistence, *RP* Rigid persistence, *OS* Occupational self-efficacy, *HP* Harmonious passion, *OP* Obsessive passion. *p < .05; **p < .01; ***p < .001. Correlations based on scale scores are reported below the diagonal; latent variable correlations from the measurement model are reported above the diagonal

**Fig. 2:**
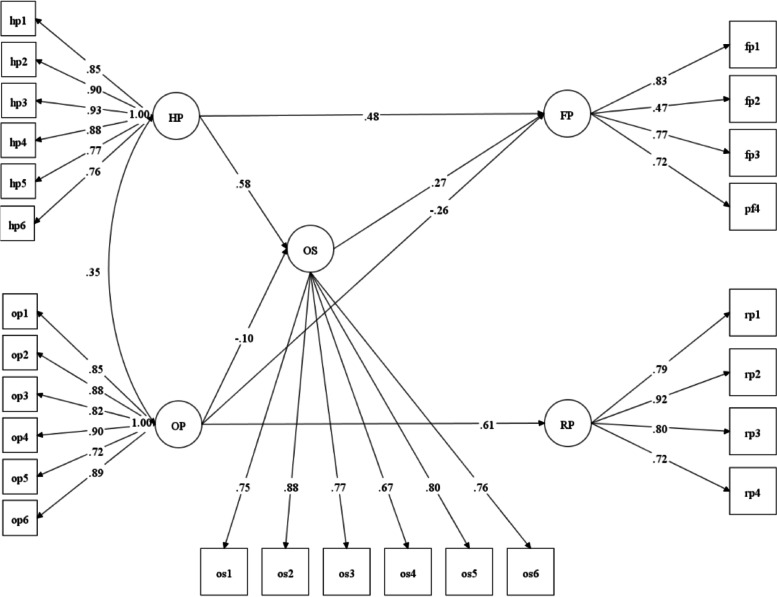
Theoretical model of passion as a predictor of persistence mediated by occupational self-efficacy. Note: *FP* = flexible persistence, *RP* = rigid persistence, *OS* = occupational self-efficacy, *HP* = harmonious passion, *OP* = obsessive passion

Structural equation modeling was performed to estimate the mediating effect of the occupational self-efficacy variable on the association between dimensions of passion and persistence. The model showed good fit indices to the available data: *χ*^2^ (df) = 891.838 (289); CFI = 0.956; TLI = 0.951; RMSEA = 0.072 90% CI = 0.067–0.078. As can be seen in Fig. 2, the path from HP to OS is positive and significant *β* = 0.580 (CI = 0.517–0.641), SE = 0.038 *p* < 0.01), whereas that between OP and OS is negative *β* =  − 0.105 (CI =  − 0.179 to − 0.017), SE = 0.049 *p* < 0.05). In turn, OS positively predicted FP *β* = 0.273 (CI = 0.177–0.368), SE = 0.058 *p* < 0.01) but not RP. In addition, direct effects were also observed. The direct effect of HP on FP was significant, *β* = 0.485 (CI = 0.329–0.579), SE = 0.057 *p* < 0.01), as was the direct effect of OP on FP *β* =  − 0.261 (CI =  − 0.347 to − 0.175), SE = 0.052 *p* < 0.01) and on RP *β* = 0.605 (CI = 0.542–0.667), SE = 0.038 *p* < 0.01). Therefore, significant mediation effects (indirect effects) were observed HP-OS-FP *β* = 0.158 (CI = 0.099–0.217), SE = 0.036 *p* < 0.01) and OP-OS-FP *β* =  − 0.027 (CI =  − 0.051 to − 0.003), SE = 0.015 *p* = 0.047).

Additionally, a significant total effect of HP on FP was observed *β* = 0.643 (CI = 0.571–0.715), SE = 0.044 *p* < 0.01) and non-significant on RP *β* = 0.063 (CI =  − 0.014–0.140), SE = 0.04 *p* = 0.181). On the contrary, there was a significant negative total effect of OP on FP *β* =  − 0.288 (CI =  − 0.376 to − 0.200), SE = 0.054 *p* < 0.01), and a significant positive total effect on RP *β* = 0.605 (CI = 0.542–0.667), SE = 0.038 *p* < 0.01). These results suggest that the occupational self-efficacy mediated approximately 24.6% of the relationship between HP and FP. and approximately 9.4% of the relationship between OP and FP. Finally, it should be highlighted that the model was able to explain 42.0% of the variance in the FP variable, 40% in RP and 31% of the variance in OS.

## Discussion

The aim of the present work was to cross-culturally adapt the RFPS to the Brazilian context and estimate the first validity evidence based on content, internal structure, reliability, and the relationship with an external variable. The results indicate that the adapted version of the RFPS (Vallerand et al., 2022) is an adequate instrument to measure the qualities of persistence in the Brazilian context (confirming H1), through two correlated factors, showing good indicators of reliability, invariance across gender and work conditions (presential vs remote), and relationships with external variables (dualistic model of passion and occupational self-efficacy) consistent with the theoretical expectations.

Regarding the internal structure, the results showed equivalence between the original and adapted instruments. Based on EFA and CFA, the two-factor model originally proposed by Vallerand et al. (2022) can be replicated and the Brazilian study presented fit indices, factor loadings, low correlations between factor, and reliability similar to those obtained in the studies of the original version in both the saturated and restricted models.

Specifically, from the EFA the four items that expressed RP were grouped into the same factor with sufficient factor loadings between, as well as adequate reliability. The results of the present research are very similar to those obtained by Vallerand et al. (2022). Additionally, the Promin oblique rotational method used in the EFA made it possible to observe a positive and weak relationship between the factors, similar to that presented in the original study with Varimax (orthogonal) rotation. Therefore, the results obtained are satisfactory, given the replication of the low magnitude correlation observed in the original version of the instrument even using a rotation method that would allow factors to correlate if a correlation exists (Damásio & Dutra, 2017; Osborne, 2015).

Considering the CFA, satisfactory factor loadings and adequate fit indices were observed. It should be noted that reliability estimates were also performed with data from this sample and that the results were satisfactory, indicating the measure’s ability to estimate the construct with a low level of associated error. Additionally, the correlation between factors was also low. Therefore, it is possible to state that there are two forms of persistence, as evidenced by the saturated model with sample 1 and by the restricted model with sample 2. The hypothesis of obtaining evidence of validity based on the internal structure and reliability (H2) of the EPRF for the Brazilian context was therefore achieved (AERA, APA, and NCME, 2014), since the factors are structured according to the theoretical proposal that guided the construction of the original instrument (Vallerand et al., 2022).

Further supporting H2, the last analysis evaluated the invariance of the measurement model in function of gender and work conditions. The results suggest RFPS strong invariancies among these groups (Millsap, 2011), corroborating those found by Chichekian and Vallerand (2022) who verified strong invariance evidence of the RFPS measure model across male and females’ students, concluding that the instrument has the same meaning for them. The knowledge on the scope for the assessment of different groups is essential for researchers involved in psychological testing, especially because the comparison between different groups should be based on empirical evidence that the observed variables (items of a test) relate to latent constructs similarly between the different groups (Milfont & Fischer, 2010).

Regarding the relationship with external variables, the theoretical hypothesis (H3) that FP would be positively correlated, with moderate magnitude, with HP and negatively, inversely correlated with OP was partially confirmed, since no statistically significant correlation was observed between FP and OP, only between FP and HP (*r* = 0.420). In fact, people with HP can fully focus on the task at hand and experience positive results both during the engagement with the activity (e.g., positive effects and flow) and afterward (e.g., feeling of satisfaction). Additionally, when prevented from engaging in passionate activity people with this type of passion are able to focus their energy and attention on other tasks that need to be performed, therefore they are people who are in control of the activity and prove to be efficient in deciding when to participate or not (Vallerand et al., 2019), which could be understood as a flexible way to persist in goals or tasks.

The hypothesis (H4) that RP would be positively correlated with both types of passion, although with greater intensity with OP was confirmed. With OP, the worker is involved in a restricted way in the activity with an uncontrollable desire to perform the task they consider important. The worker cannot stop doing or thinking about the passionate activity because it dominates him or her. As such, OP leads the worker to persist rigidly on work goals. Although this rigid persistence in performing the activity can have beneficial results, such as high performance, it can also generate conflicts with other goals since frustrations and ruminative thoughts can accompany the individual when they are not involved in the passionate activity (Vallerand et al., 2019).

Finally, the conceptual model (H5) in which HP would positively predict FP, and OP would negatively predict FP and positively predict RP was confirmed. These results suggested that the way someone engages in a task that they are passionate about dictates the quality of that persistence, therefore, HP can lead to the development of FP, which allows the individual to persist in different goals and activities as well as the main one in a more pleasant and consonant way. On the other hand, obsessive passion is capable of providing a more restricted type of action in the involvement with the activity, being positively associated with RP and negatively associated with FP (Vallerand et al., 2022).

Similar results were observed by Chichekian and Vallerand (2022) when applying the dualistic model of passion and persistence in the educational context. With a sample of 591 Canadian students (357 women) they verified the explanatory power of HP and OP on FP and RP. Harmonious Passion was able to positively predict FP, while Obsessive Passion was positively associated with RP and negatively associated with FP. These findings demonstrate that both types of persistence were empirically associated with HP and OP.

Finally, it was possible to show through the mediation model (H6) occupational self-efficacy partially mediated the relationship between HP and OP on FP. This corroborates the theoretical expectation, since as professional experience allows the internalization of the professional activity and identity in a harmonious way the individual tends to present higher levels of engagement and performance at work (Houlfort et al., 2014; Vallerand et al., 2019), as well as the experience of more positive emotions during and after professional activities (Bélanger et al., 2014; Slemp et al., 2021). This leads to the development of beliefs that they are able to adequately perform their professional activities (Pollack et al., 2020), and therefore to establish a flexible persistence relationship with this activity (Vallerand et al., 2022).

These results are similar to those obtained by Feng and Chen (2020) with a sample of Japanese entrepreneurs where passion for entrepreneurship contributed positively to entrepreneurial persistence behavior and entrepreneurial self-efficacy which in turn also positively affected persistence. This would attribute a mediating role of self-efficacy in the relationship between HP and persistence helping subjects to improve the recognition of their entrepreneurial role, maintaining an optimistic and positive attitude in the face of professional challenges and stimulating creativity and innovation to maintain entrepreneurial persistence.

The protective role that occupational self-efficacy assumed in the present study in the relationship between OP and FP should be emphasized. This indicates that workers who are passionate about work tend to have difficulty perceiving themselves as effective in those activities given the internal and external pressures that characterize this type of involvement with the activity (Vallerand et al., 2019) and the negative emotions in the work context, such as psychological distress and burnout (Slemp et al., 2021). Given this scenario, the perception of self-efficacy can mitigate the direct negative relationship between OP and FP, that is, workers with obsessive passion when perceiving themselves as more effectively capable of the professional activities can establish flexible persistence in relation to this passionate activity being able, for example, to enjoy positive experiences in other areas of life. These results can be truly relevant for the development of interventions that aim to increase self-efficacy (Ouweneel et al., 2013) with professionals who have high levels of OP.

The results of the present research suggest important practical implications for the constructs studied. It empathizes a supportive organizational structure where pressure and ego-involvement are minimized facilitating harmonious engagement with the work activity and a flexible persistence in this activity execution. Additionally, using the findings of this study, strategies can be promoted and implemented for developing professional intrapersonal knowledge. This type o knowledge may involve understanding the ways of engagement in the professional activity and the consequences of each type of engagement, as well as tools to achieve this goal. Thus, the support of psychology professionals for the workers could be helpful to the improvement of their occupational self-efficacy perception and flexible relationship with work activity, which could promote personal and organizations well-being.

Finally, some limitations of the study should be underscored. The data collected and analyzed came from a convenience sample during the COVID-19 pandemic. Accordingly, studies with other samples should be performed outside the pandemic context. Another important limitation concerns the use of a cross-sectional research design to test the associations between the variables used in the study, which prevents the inference of causality among the variables. Therefore, new studies based on longitudinal and experimental designs should be carried out to assess the replicability of these results and to better understand how these variables interact over time. Based on this idea, the assessment of test–retest reliability should be investigated. It is worth mentioning the potential of response biases (e.g., social desirability, acquiescence) on self-report instruments, especially in high stakes contexts. In this sense, it is suggested the use of methods that could mitigate the effects of these response biases. Research investment is also recommended to estimate the instrument’s potential using other psychometrics methods, such as item response theory, with the goal of evaluating the difficulty and discrimination properties of the items, as well as the establishment of interpretative norms that allow the attribution of psychological meaning to the RFPS scores. Lastly, the psychometric properties of the RFPS can be assessed within the context of other activities besides work, such as education and sports.

## Conclusions

This study made it possible to confirm the potential of the Brazilian Portuguese version of the EPRF to assess Rigid and Flexible Persistence in the Brazilian population by demonstrating the stability of the instrument as well as the internal structure, reliability, generalizability across group and relationship with external variables. The findings of this first study provide support for the psychometric properties of the Brazilian version of the RFPS and stimulate the continuity of research into the construct of persistence using this instrument in future studies in order to assess the consequences of the two dimensions of persistence and the replication of results found in this study, what will contribute to the accumulation of validity evidence to support the interpretation of the RFPS scores.

## Data Availability

The datasets used and/or analyzed during the current study are available from the corresponding author on reasonable request.

## Abbreviations

CFA: Confirmatory factor analysis
CFI: Comparative fit index
CI: Confidence interval
CVC: Content validity coefficient
EFA: Exploratory factor analysis
FP: Flexible persistence
HP: Harmonious passion
LC: Language clarity
OP: Obsessive passion
DF: Degrees of freedom
OSS-SF: *Occupational Self-efficacy* *Scale* *–Short form*
RF: Rigid persistence
RFPS: Rigid and Flexible Persistence Scale
RMSEA: Root mean square error of approximation
SE: Standard errors
TD: Theoretical dimension
TLI: Tucker–Lewis index
TR: Theoretical relevance
WLSMV: Weighted least squares mean and variances adjusted
